# Dyskeratosis Congenita: A Case Report of a Patient With Coronary Artery Disease

**DOI:** 10.7759/cureus.40939

**Published:** 2023-06-25

**Authors:** Michael Ghaly, Mark Ghaly, Samuel Harris

**Affiliations:** 1 Internal Medicine, Baptist Memorial Hospital, Oxford, USA; 2 Biology, Georgia Southern University, Savannah, USA

**Keywords:** pulmonary fibrosis, cardiovascular disease (cvd), cardiovascular prevention, aplastic anemia, st-elevation myocardial infarction (stemi), coronary artery by-pass grafting, telomere shortening, heart failure with reduced ejection fraction, dyskeratosis congenita, coronary artery disease

## Abstract

Clinical evidence demonstrates that patients with telomere biology disorders, such as dyskeratosis congenita, are more prone to coronary artery disease. We present the case of a 43-year-old female diagnosed with dyskeratosis congenita with critical cardiovascular disease. She underwent coronary artery bypass graft (CABG) with improvement of her cardiac function. Although this is a rare genetic disease, further studies are warranted to investigate the underlying pathophysiology of cardiovascular disease in patients with dyskeratosis congenita.

## Introduction

Cardiovascular disease is one of the leading causes of death. Some of the common risk factors that contribute to cardiovascular disease include hypertension, diabetes, physical inactivity, poor diet, and tobacco use [1]. Biomarkers are also used to assess for coronary artery disease (CAD), including serum low-density lipoprotein (LDL), cholesterol, triglycerides (TG), lipoprotein (a), high sensitivity C-reactive protein (hs-CRP) and homocysteine [1].

In the field of preventive cardiology, research focuses on early recognition, diagnosis, and treatment of the disorder using these various biomarkers. Recent studies demonstrated that patients with shortened telomeres are affected with CAD [2]. Dyskeratosis congenita (DC), also known as Zinsser Engman-Cole syndrome or short telomere disease, is a rare disorder that can be characterized by unique findings including fingernail and toenail dystrophy, reticular pigmentation of the upper torso and neck and oral leukoplakia [3]. It is inherited in an X-linked recessive pattern that is caused by mutations that interfere with normal maintenance of telomeres, the regions that protect the loss of genetic material [3]. DC is also directly related to bone marrow failure and cancer predisposition syndrome, which arises from flaws in telomere sequence maintenance [3]. Numerous individuals diagnosed with DC have also been found to be diagnosed with CAD, aplastic anemia, idiopathic pulmonary fibrosis, cirrhosis or sporadic congenital abnormalities. It is estimated to be found in every one out of 1 million people [4]. Given this evidence, we present the case of a patient diagnosed with DC with significant CAD.

## Case presentation

**Figure 1:**
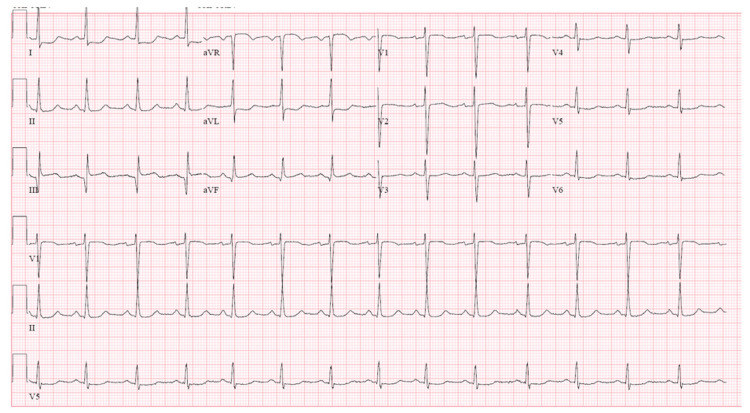
EKG findings showed ST segment elevation in lead III as well as diffuse ST segment depression

Our patient is a 43-year-old female with past medical history of aplastic anemia, dyskeratosis congenita, and pulmonary fibrosis with chronic hypoxic respiratory failure. She presented to our emergency department with complaints of exertional chest pain for a duration of one week. She has a remote history of smoking. The patient’s family history was significant for dyskeratosis congenita including pulmonary fibrosis in her mother who passed away in her 50s, maternal aunt and uncle, and her cousins. Coronary artery disease was also present in her aunt. The patient denied any palpitations, presyncopal or syncopal episodes, orthopnea, paroxysmal nocturnal dyspnea, and no history of deep vein thrombosis or embolism. She reported that as she was doing her household chores, she experienced sudden fatigue and shortness of breath, prompting her to present to the emergency department. On presentation, an electrocardiogram (EKG) showed diffuse ST segment depressions. There was also an ST elevation in lead III however, it did not meet criteria for ST-elevation myocardial infarction (STEMI) as there was no elevation in contiguous leads II or aVF. There was also elevation in lead V1 but not V2 (Figure 1). Troponin was elevated at 0.4 and trended upwards to 1.19. 

**Figure 2:**
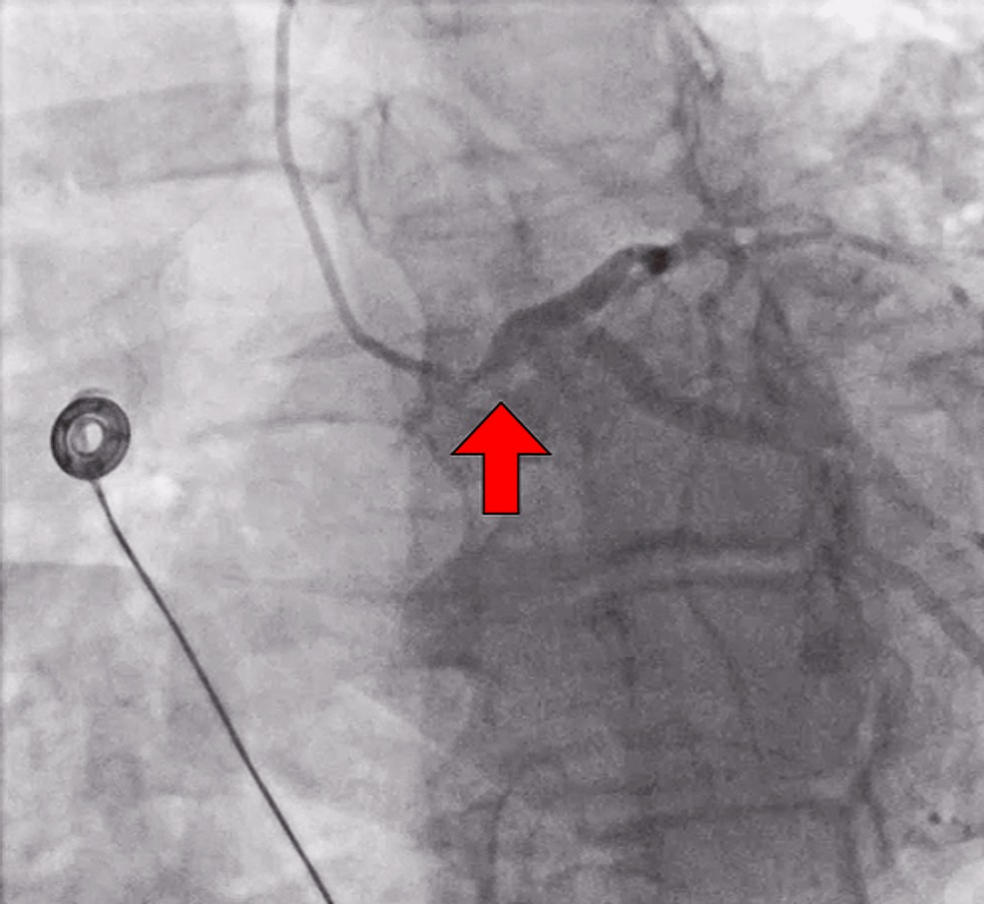
Left main coronary ostial stenosis Critical left main ostial stenosis (red arrow)

**Figure 3:**
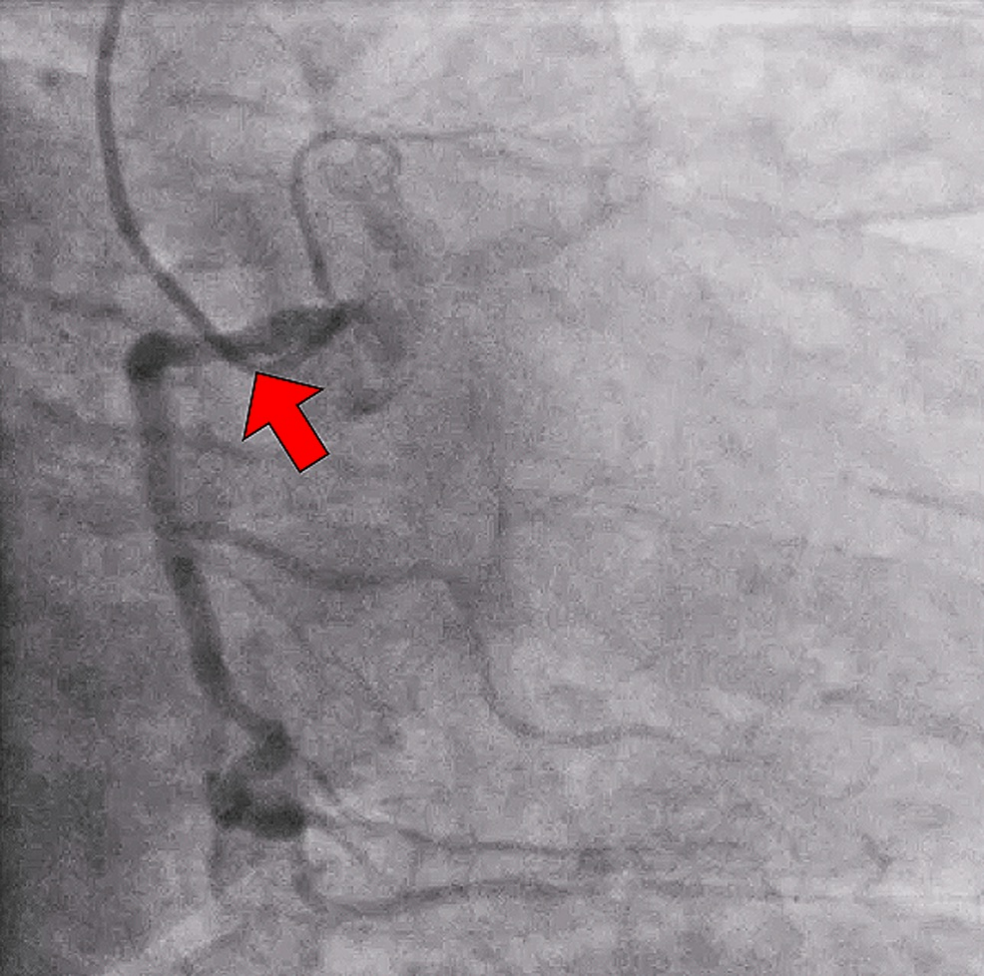
Right coronary artery Right coronary artery showed approximately 50% stenosis (red arrow)

The patient was taken to the cardiac catheterization lab and was found to have critical left main disease with 95% ostial stenosis (Figure 2). The left anterior descending artery and left circumflex showed luminal irregularities up to 25%. The right coronary artery had 50% stenosis (Figure 3). An impella was placed through her right common femoral artery and she was transferred to the intensive care unit. Approximately four hours later, the patient began experiencing chest pain again and she was taken emergently to the operating room for coronary artery bypass graft (CABG) with left internal mammary artery to the left ascending artery and the right saphenous vein graft to the obtuse marginal 2. The impella was subsequently removed through the right common femoral artery. The patient tolerated the surgery and was monitored in the intensive care unit postoperatively. The patient had a prolonged intubation due to volume overload from cardiogenic shock. She was diuresed with furosemide. Vasopressors were weaned as tolerated. Patient then underwent direct current cardioversion by cardiology and was placed on an amiodarone infusion for rhythm control after developing atrial fibrillation. She was started on apixaban. Her sternum incisions were well approximated and intact. Patient was discharged and advised to follow up with cardiothoracic surgery as an outpatient.

Prior to her CABG, her echocardiogram showed severe hypokinesis of the left ventricle. The ejection fraction (EF) range was estimated between 20 to 25%. She also had mild mitral regurgitation. A few weeks after her procedure, a transesophageal echo showed a ventricular systolic function to 30%. There was also a small patent foramen ovale noted with minimal left-to-right shunting. She then had another cardiology follow-up appointment several months later which showed an improvement of her EF to 40 to 50%.

A high-resolution computed tomography (CT) scan reported diffuse chronic interstitial thickening, as well as, patchy ground glass opacities in both lungs with mild cylindrical bronchiectasis in both lungs. Pulmonary function tests (PFTs) were done several months prior which showed forced expiratory volume (FEV1) to forced vital capacity (FVC) ratio of 69%. Total lung capacity was diminished at 45% of predicted. Residual volume is also diminished at 36% predicted. PFTs were conclusive for moderate restrictive lung disease. The patient was then scheduled for a lung biopsy. Lung wedge biopsies from the right upper and middle lobes showed interstitial lung disease. In most areas, the interstitial changes are relatively diffuse and uniform resembling those seen in the so-called fibrotic nonspecific interstitial pneumonia (NSIP). Focally, there were areas showing fibrosis with early microscopic honeycomb change and scattered subepithelial fibroblast foci. These findings are suggestive of usual interstitial pneumonia (UIP). Within the air spaces, there were also clusters of lightly pigmented alveolar macrophages diagnostic of respiratory bronchiolitis, a finding that is seen most commonly in cigarette smokers. The patient followed up with pulmonary rehabilitation and was referred to a lung transplant center.

## Discussion

Although DC is primarily recognized for hematological and malignant complications, some evidence suggests that individuals with DC may also be at an increased risk of developing cardiovascular disease, particularly CAD [5].

We present the case of a middle-aged female with DC who presented with symptoms of CAD. She had no common risk factors for CAD, such as hypertension, diabetes, hyperlipidemia, or obesity. She ate a healthy diet and did not have a sedentary lifestyle, although her exercise was limited due to her pulmonary fibrosis. We believe that her smoking history was non-contributory as it was remote. She also had a family member with no risk factors who was a non-smoker and also developed CAD. The diagnosis of CAD secondary to DC was made based on her clinical presentation and angiographic findings.

While there have been only a few reports of CAD in patients with DC, there is increasing evidence to suggest that DC may be a risk factor for cardiovascular disease [5]. A few studies have shown that patients with DC have increased arterial stiffness as well as endothelial oxidative damage [5]. The mechanism by which DC predisposes to CAD is not completely understood. However, it has been hypothesized that the underlying telomere dysfunction may impair vascular repair mechanisms and increase susceptibility to atherosclerosis. In patients with normal telomerase activity, approximately 50 to 100 base pairs are lost with each cell division. There are several telomerase regulator genes (TERT, TERC, DKC1, TINF2) that may be mutated in patients with telomere biology disorders [5]. Therefore, affected individuals can lose up to twice as many base pairs compared to the average individual annually. Several factors can cause telomeres to be shortened. For instance, aging, degenerative disease, and psychological stress are contributing regulators of telomere length [6]. Additionally, atherosclerosis, insulin resistance, and diabetes are associated with telomere shortening. As a result of low telomerase activity, the hypothalamus, pituitary, adrenal activation increases glucocorticoid production in response to stress. Subsequently, this leads to production of reactive oxygen species (ROS) levels and inflammatory mediators which increase the susceptibility for age-related disease, including cardiovascular disease [6].

This case highlights the importance of early screening and preventive measures in patients with DC, including management of regular cardiovascular risk factors including hypertension, lipids, and diabetes.

## Conclusions

In conclusion, our patient has a rare genetic disorder associated with a range of medical complications, including bone marrow failure, pulmonary fibrosis, and coronary artery disease. Although, the exact pathophysiological mechanism underlying the association between dyskeratosis congenita and coronary artery disease remains unclear, it is important to consider this possibility in patients with symptoms suggestive of cardiovascular disease. To prevent adverse outcomes, early diagnosis and management are important by controlling risk factors as well as monitoring for potential cardiovascular complications. It often requires multidisciplinary collaboration between hematologists, cardiologists, pulmonologists, and other medical specialties to optimize patient outcomes. Further research is needed to better understand the underlying mechanisms and risk factors for cardiovascular disease in patients with dyskeratosis congenita.
